# Acoustofluidic Micromixing Enabled Hybrid Integrated Colorimetric Sensing, for Rapid Point-of-Care Measurement of Salivary Potassium

**DOI:** 10.3390/bios9020073

**Published:** 2019-05-28

**Authors:** Vikram Surendran, Thomas Chiulli, Swetha Manoharan, Stephen Knisley, Muthukumaran Packirisamy, Arvind Chandrasekaran

**Affiliations:** 1 Department of Chemical, Biological and Bio Engineering, North Carolina A & T State University, Greensboro, NC 27411, USA; vsurendran@aggies.ncat.edu (V.S.); tjchiulli@aggies.ncat.edu (T.C.); smanoharan@aggies.ncat.edu (S.M.); sbknisle@ncat.edu (S.K.); 2 Department of Mechanical, Industrial and Aerospace Engineering, Concordia University, Montreal, QC H2L5C9, Canada; pmuthu@alcor.concordia.ca

**Keywords:** point-of-care, biosensor, hybrid integration, microfluidics, acoustofluidics, cavitation, micromixing, optical absorbance, colorimetry, salivary potassium

## Abstract

The integration of microfluidics with advanced biosensor technologies offers tremendous advantages such as smaller sample volume requirement and precise handling of samples and reagents, for developing affordable point-of-care testing methodologies that could be used in hospitals for monitoring patients. However, the success and popularity of point-of-care diagnosis lies with the generation of instantaneous and reliable results through in situ tests conducted in a painless, non-invasive manner. This work presents the development of a simple, hybrid integrated optical microfluidic biosensor for rapid detection of analytes in test samples. The proposed biosensor works on the principle of colorimetric optical absorption, wherein samples mixed with suitable chromogenic substrates induce a color change dependent upon the analyte concentration that could then be detected by the absorbance of light in its path length. This optical detection scheme has been hybrid integrated with an acoustofluidic micromixing unit to enable uniform mixing of fluids within the device. As a proof-of-concept, we have demonstrated the real-time application of our biosensor format for the detection of potassium in whole saliva samples. The results show that our lab-on-a-chip technology could provide a useful strategy in biomedical diagnoses for rapid analyte detection towards clinical point-of-care testing applications.

## 1. Introduction

Point-of-care diagnostics (POCD) for health monitoring involves the evaluation of indices from human health through tests performed outside of the clinical laboratory, typically right at the site of patient care [1,2,3,4]. While the advantages of POCD such as rapid diagnosis, operational efficiency, and costs have been well established [2], widespread implementation of this technology has not yet been achieved [5]. One of the main limitations of POCD is the requirement of unprocessed test samples drawn from patients to provide the desired sensitivity and specificity for conducting reliable assays in situ. However, most of the currently validated clinical assays are carried out using plasma, prepared from whole blood samples inside sufficiently equipped laboratory conditions. Here, we develop a biosensor platform that could be reliably used for instantaneous detection of target molecules from non-invasively obtained bodily fluid samples for real-time point-of-care testing applications.

Although whole blood assays are generally considered as the gold standard for bioanalysis, such an approach may not be convenient to implement under all circumstances. In general, high-risk patients under intensive care or long-term hospitalization suffering from extremely sick conditions may not always be able to bleed enough quantity of blood. Possible nerve damage during venipuncture [6] could render the procedure extremely painful, traumatic and psychologically disturbing for the patients, such that even skilled phlebotomists could face limitations to extract enough quantity of blood under such circumstances.

One strategy to overcome the trauma associated with such painful venous blood draw procedures could be to adopt a technique that is less invasive and utilizes smaller volumes, possibly through a finger prick as implemented with some of the commercially available handheld blood glucose monitors. However, a limitation with this approach is that reduced sample volumes call for greater sensitivity of the measurement setup and may also not always contain the necessary distribution of the target molecules under all circumstances. This could lead to inconsistent readings and false negative results [7]. Furthermore, common clinical problems with whole blood assays such as hemolysis or coagulation could also result in an inaccurate representation of the analyte concentration [8].

**Figure 1:**
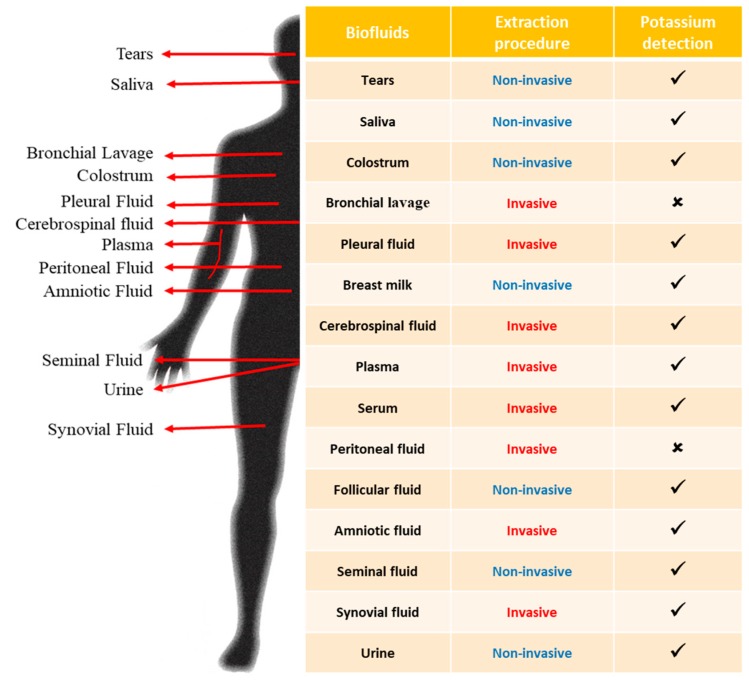
List of commonly available bodily fluids showing their nature of the extraction procedure and general feasibility of potassium detection from those fluids.

An alternate strategy would be to eliminate the necessity for whole blood, and use other test samples for carrying out the required bioanalysis. Among the bodily fluids generally used in clinical assays (shown in Figure 1), those commonly available through non-invasive extraction procedures include urine, saliva [9] and sweat [10]. While inducing sweat out of hospitalized patients is practically difficult, urinary extraction may also necessitate additional diuretics which could directly interfere with the measurands, aside from inducing other significant side effects [11]. Considering these factors, saliva presents a viable alternate to the traditional biofluids used in biochemical analyses.

Saliva is considered as a filtrate of the blood representing the physiological state of the body. Given the ability of molecules from blood to diffuse into saliva, salivary diagnostics is increasingly being recognized as an equivalent to serum analysis [12]. Research studies using saliva as a bio-diagnostic fluid includes works such as evaluation towards dialysis needs in renal failure patients [9] and on-site analysis for biochemical factors [13]. The incorporation of microfluidic methods in bioassays of saliva has further reduced sample and reagent consumption, and has decreased the overall assay times [13]. Research studies in salivary diagnostics involving microfluidics include measurement of C-reactive protein using an fluorometric immunoassay [14], on-chip polymerase chain reaction (PCR) system for rapid fluorometric detection of genetic deletion [15], paper-based device for quantification of the nitrate concentration [16], detection of thiocyanate through surface-enhanced Raman scattering (SERS) [17], and spectrometric absorbance detection of NH_3_ and CO_2_ in saliva as a biomarker for stomach cancer [18]. In addition, most of the reported microfluidic systems for salivary diagnostics included integrated optical sensing for their bio-detection scheme [19].

In this work, we present the development of a simple, hybrid integrated optical microfluidic biosensor for rapid analysis of saliva, and we have demonstrated the application of the proposed biosensor format by detecting the presence of potassium from whole, untreated salivary samples. Inspired by the age-old concept of colorimetry published as early as the first half of the 20th century [20,21], we hereby show that potassium can be detected from human saliva samples through the principle of optical absorption. A commercially available potassium detection reagent mixed with saliva samples creates turbidity based on the potassium concentration, which could then be measured through the absorption of light passing through this turbid path. We have implemented this optical sensing principle on a lab-on-a-chip platform by integrating colorimetric detection within a microfluidic system that facilitates transportation and handling of the analytes and the reagents. Homogeneous mixing of fluids inside the microfluidic system has been achieved through the integration of piezo-actuated acoustic micromixing. The results of this work show that the proposed hybrid integrated device can be applied to real-time optical biosensing using whole, unprocessed samples, which could be extremely useful for automated point-of-care testing applications.

## 2. Materials and Methods

### 2.1. Development of the Integrated Optical Biosensor

**Figure 2:**
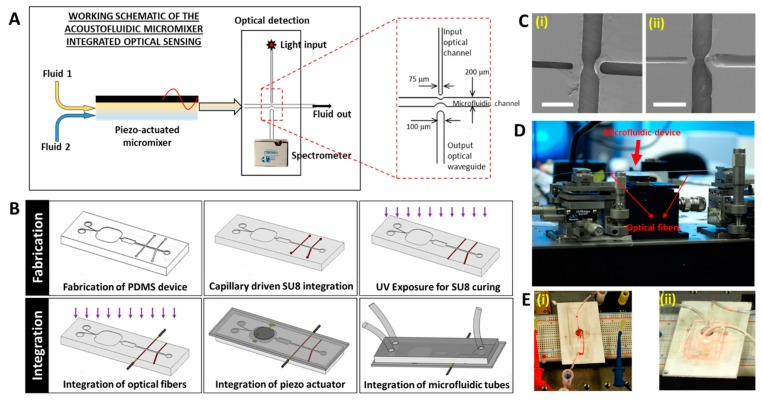
(**A**) Schematic working principle of the acoustofluidic micromixer integrated optical sensing for colorimetric detections. The liquid test specimen (fluid 1) and its corresponding chromogenic agent (fluid 2) are mixed using acoustic waves generated by a piezoacuator, and the mixture is transported into the optical detection unit, wherein the extent of color change induced due to the presence of target analyte in the sample is sensed by the absorption of light passing through the sample. (**B**) Schematic illustrayion of the fabrication and packaging of the hybrid integrated biosensor (**C**) Scanning electron microscope (SEM) images showing the microfluidic channel and the optical channels of the poly dimethylsiloxane (PDMS) device (i) before and (ii) after integration with SU8 (Scale bars represent 500 m) (**D**) Micro-positioning setup for precise alignment and coupling of fibers with the SU8 waveguides. (**E**) Images of the functional integrated optical microfluidic biosensor (i) with one fluid inlet and (ii) two fluid inlets.

This section provides the details of the design, fabrication, integration and packaging of our hybrid integrated optical microfluidic setup. The schematic illustration of the processes is shown in Figure 2.

#### 2.1.1. Device Design and Fabrication

The biosensor (schematically shown in Figure 2A) consists of a microfluidic system with two inlets which accommodates two different types of fluids. The fluids are transported to a piezo-actuated (acoustofluidic) micromixer unit, and upon mixing are subsequently transported to the optical detection unit. The configuration of the bulk acoustofluidic micromixer unit was adopted from [22,23]. The optical detection unit consists of SU8 waveguides core integrated onto poly dimethylsiloxane (PDMS) (cladding) through the inlet and outlet waveguide channels (as indicated in Figure 2A). Convex curvatures at the end of the optical channel enable convergence of light into the fluidic channel. Two different types of optical waveguide assembly were designed. In the configuration used in the present work, the output waveguide was fabricated co-axially with the input waveguide. This configuration allowed direct coupling of the input light onto the output waveguide, as required in optical absorption measurements.

To fabricate the devices on PDMS (fabrication and integration processes schematically shown in Figure 2B), silicon master molds with patterned SU8 (100 μm thickness) were fabricated using standard soft lithography process. Poly dimethylsiloxane (Sylgard 184, Dow Corning, Midland, MI, USA) with prepolymer to curing agent volumetric ratio of 10:1 was mixed, degassed and poured onto the silicon mold. The setup was left to cure at 70 °C for four hours, after which PDMS was peeled off the mold and diced to create the individual devices. To create optical waveguides, the PDMS devices were exposed to oxygen plasma (2 min, 200 mTorr pressure, 20 cubic centimeter (ccm) flow rate, and 60 W Radio Frequency (RF) power), and thereafter SU8-5 (Microchem, Westborough, MA, USA) with viscosity 290 cSt, and refractive index ~1.6 was allowed to fill in the optical channels through capillary flow. The photoresist was cured by flood exposure to ultraviolet (UV) light (12 mW/cm^2^) for 60 s, and the devices were diced thereafter to enable fiber attachment with the SU8 waveguides.

#### 2.1.2. Integration and Packaging

Coupling of the input and the output fibers were carried out under a stereo microscope. FC connectorized tapered lens-ended fiber (OZ Optics, Ottawa, ON, Canada) which gives a spot size of 5 μm, was connected to a broadband laser light source (Ocean Optics, Largo, FL, USA) for optical input. The output collector fiber was SMA end connectorized and coupled to a handheld spectrometer (USB 2000, Ocean Optics, Largo, FL, USA). Fiber strippers and precision cleavers (Newport, RI, USA) were used to remove the buffer layer and cladding around the fiber. The input and the output fibers were positioned in separate five axis fiber positioners on supporting V-grooves, so that each of the fibers and the waveguides can be maneuvered and coupled appropriately for acquiring the maximum optical signal. Maximum optical light coupling into the fiber was ensured by fine-tuning the position of the fiber and by observing the maximum signal from the output fiber coupled into the spectrometer. Thereafter, UV index matching gel (NOA60, Norland International Inc., Lincoln, NE, USA) was applied at the tip of the fiber and the setup was exposed to UV for 60 s to bond the fiber with the SU8 waveguides.

The device was then treated with atmospheric plasma using a handheld plasma cleaner (Plasmaetch, Carson City, NV, USA) and the channels were sealed using a 100 μm thick glass coverslip. Piezoceramic discs (T216-A4NO-173X, Piezo Systems Inc., Cambridge, MA, USA) used for generating the acoustic waves were attached to this glass diaphragm using silver conductive epoxy (also used as the bottom electrode). The top electrode was soldered to the piezoceramic. For ease of handling, the device was attached with a polycarbonate support using double-sided adhesive tape, without damaging the optical fibers. A through hole drilled on the polycarbonate prior to the attachment of the optical-microfluidic chip accomomodates the piezo actuator inside the cavity.

### 2.2. Chemicals and Reagents

Integrated optical-microfluidic characterization experiments were carried out with de-ionized (DI) water and ethanol (Sigma Aldrich, St. Louis, MO, USA). Prior to introducing fluid samples into the system, the microfluidic channels were flushed with ethanol in order to remove any contaminants or air bubbles. Initial micromixing characterization experiments were conducted by staining the working fluids (DI water or ethanol) with standard food coloring dyes. Preliminary experiments for the characterization of optical absorption with integrated micromixing were conducted using glucose enriched RPMI 1640 cell culture media (A1049101, Thermofisher, Waltham, MA, USA) as the working fluid. Herein, the glucose present in cell culture media was tested to produce an expected colorimetric reaction with Benedict’s reagent prepared according to the protocol described by Cochran et al. [24]. For colorimetric salivary analysis experiments, potassium colorimetric assay kit (E-BC-K279) was procured from Cedarlane Labs (Burlington, NC, USA). Whole samples of unstimulated saliva were collected from volunteers in centrifuge tubes by a simple spitting method, and the specimens were vortexed thoroughly. Prior to the experiments, protein precipitant and chromogenic agents were prepared as described in the protocol prescribed by the manufacturer. 20 µL of the saliva samples were mixed with 180 µL of the protein precipitant and centrifuged at 1100 g for 10 min. Thereafter, the supernatant was used in the experiments, mixed with a volumetrically consistent chromogenic agent for the colorimetric optical absorbance measurements.

### 2.3. Integrated Testing and Measurement

The piezoelectric system was driven by an external signal generator (33120A, Hewlett Packard, Palo Alto, CA, USA) and an amplifier (PCB Piezotronics, Depew, NY, USA) where a sinusoidal signal from the signal generator was amplified 20-fold by the amplifier. Typically, the operating voltage of 100 V_p-p_ was used for piezo-actuation. For continuous flow experiments, liquids were injected at the same flow rates inside the microchannel using syringe pump (KDS-210, KD Scientific, Holliston, MA, USA). In all other cases, liquids were manually pipetted into the microfluidic channels.

The mixing efficiency was calculated based on the change in the pixel values of the respective color fluids.

Mixing efficiency (%) = (Initial pixel count-final pixel count)/(Initial pixel count) × 100(1)

Optical signals were collected using a handheld spectrometer (USB2000, Ocean Optics, USA). The optical absorbance of the specimens measured by the spectrometer is given by the formula

(2) $$A_{\lambda }=\;-log_{10}\left(\frac{S_{\lambda }-D_{\lambda }}{R_{\lambda }-D_{\lambda }}\right)$$

where,

*λ*—Wavelength of light used
*A_λ_*—Absorbance
*S_λ_*—Intensity of light passing through the sample
*D_λ_*—Dark intensity
*R_λ_*—Intensity of light passing through a reference medium.

The dark intensity was recorded by measuring the optical signal intensity when the light source was turned off. For potassium measurement experiments, the chromogenic agent was used as the reference medium to measure the reference intensity.

### 2.4. Imaging and Statistical Analysis

Images were recorded using an OFV-A-534-Cax video camera inbuilt with the single point Laser Doppler Vibrometer (LDV, Polytec, Detroit, MI, USA). Scanning electron microscopy (SEM, SU3500, Hitachi Hi-Technologies, Tokyo, Japan) was conducted using standard protocols for variable-pressure imaging mode (3.0 kV, 30 Pa,) allowed SEM observations of the PDMS devices without the need for additional sample manipulation or conductive coating. The images were processed using ImageJ (National Institute of Health, Bethesda, MD, USA), following standard protocols. All statistical comparisons were made using one- or two-way analyses of variance (ANOVA) with Tukey post-hoc comparisons (Prism; GraphPad Software, La Jolla, CA) with *p*-values < 0.05 considered significant, and graphical data reported as means ± standard error for at least n = 3 experiments.

## 3. Results

### 3.1. Features of the Hybrid Integrated Biosensor

The SU8 waveguide (refractive Index ~1.6) integrated well onto the PDMS (refractive index ~1.4) upon curing with UV (Figure 2C). Minor delamination of the SU8 was observed along the walls of the PDMS in some sections, however SU8 was able to guide the input light efficiently under the PDMS clad. The optical fiber assembly setup (Figure 2D) described earlier provides robust fiber attachment onto the waveguides to enable optical transmission. Also, with this arrangement of the optical microfluidic chip, the microfluidics and the micromixer modules do not interfere with the functionalities of the optical ensembles, and thus all the components of the system remain independent. Integration of on-chip spectrometer [25] was also tested to conduct direct fluorescence measurement that could be used for specific immunoassays. The fully fabricated hybrid integrated optical microfluidic device is as shown in Figure 2E.

Initial vibration analysis experiments for the mechanical characterization the piezo-actuation system was conducted using laser doppler vibrometry. The natural frequency of oscillation of the piezo-actuator was ~70 kHz, much higher than the intended operating frequencies of the micromixer. As expected, the acoustic waveform generated by the piezo-actuation depends on the operating frequency. For this device configuration, acoustic cavitation inception occurred for operating frequencies greater than ~800 Hz. Upon cavitation inception, the rate of cavitation bubble growth and collapse was dependent on the operating frequency, and these parameters stabilized at ~3000 Hz, beyond which no significant differences were observed in the life-cycle or the behavior of the cavitation bubbles. On the other hand, at lower operating frequencies (1~100 Hz), the piezo-actuation principle could be used for transporting fluid across the channels through valveless micropumping [24]. Slight modifications to the existing design enable the feasibility of integrating other types of optical detection techniques, namely fluorescence or evanescence for a correspondingly appropriate type of integrated microfluidic biosensing [26].

### 3.2. Acoustically Induced Cavitation Enables Rapid Micromixing

Effective mixing of fluids is important in several applications including chemical synthesis, biochemical reactions, and clinical diagnosis. As with any biochemical assay, for accurate colorimetric quantification sufficient mixing of fluids is important in order to homogenize the specimens with the corresponding reagents [27]. However, one of the limitations with microfluidics is the laminarity of flow in the device, which renders turbulent mixing difficult. Here, by incorporating a piezo-actuated acoustic micromixer within the microfluidic design, we experimentally demonstrated the feasibility of controlled mixing of two non-reacting liquids through the inception of cavitation bubbles.

**Figure 3:**
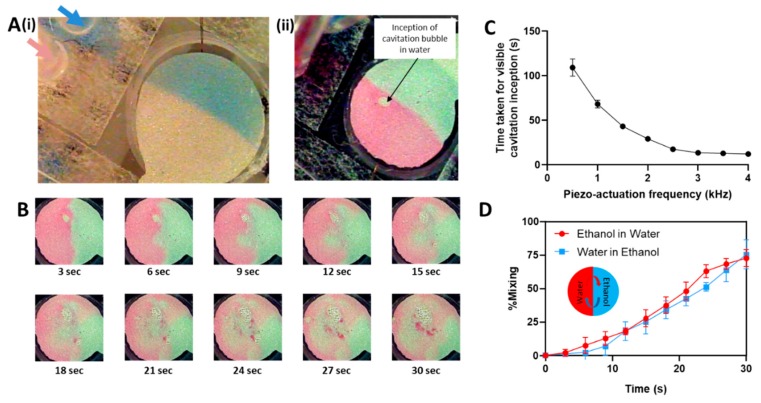
(**A**) (i) Image of the microfluidic channel with two inlets allowing laminar flow of two shades of ethanol (colorless and blue) without mixing when introduced from either of the inlets. (ii) Image showing the inception of cavitation bubble in water (red) to initiate the mixing of fluids (**B**) Image sequence showing the cavitation enabled mixing of water and ethanol inside the microfluidic channel. (**C**) Variation of the time taken for the inception of a visible cavitation bubble with respect to the piezo actuation frequency. Cavitation inception time was consistent (within 3% variation) for actuation frequencies greater than 1 kHz. (**D**) variation in the fraction of the respective fluids mixed inside the microchannel with respect to time. To enable this fluid mixing, caviation was initiated at 1000 Hz actuation frequency, and the working frequency was reduced to 800 Hz, so as to retard the growth of the cavitation bubble.

For convenience and ease of visualization, ethanol was stained blue and water was stained with red dye in all the experiments. Under continuous flow, the flow of liquids inside the microfluidic channel was extremely laminar (calculated Reynolds number ~5). The mixing ability of fluids under flow was tested in different conditions, and no mixing was observed even for similar liquids of very high miscibility (blue ethanol colorless ethanol) in both with or without the actuation of the micromixer (Figure 3A(i)). For dissimilar liquids (water–ethanol), instantaneous diffusion mixing was also negligible for both continuous flow and ‘static’ (no flow) conditions. Once the flow of liquid in the channel was stopped, actuation of the micromixer at 1000 Hz induced the formation of cavitation bubble in water (Figure 3A(ii)). We believe that PDMS substrates, being gas permeable facilitates the generation of bubbles, even for such low actuation frequencies, which in this case was useful for fluid mixing. Thereafter, reduction of the operating frequency to 800 Hz retarded the growth of the cavitation bubble, and at the same time creating micro-vortices in the fluids to initiate mixing. The real-time sequence of cavitation assisted fluid mixing is as shown in Figure 3B. Mixing of the fluids was instantaneous upon the inception of the cavitation bubble. Increasing the actuation frequency decreased the time taken for cavitation bubble inception (Figure 3C). However, with increased frequency, the growth of the cavitation bubble was also rapid and difficult to control precisely. Therefore, to create steady cavitation, all micromixing experiments were conducted at an actuation frequency of 1000 Hz to initiate the cavitation bubble, and thereafter the operating frequency was reduced to 800 Hz to allow fluid mixing. The actuation frequency of our bulk acoustofluidic micromixer is around 100 fold lower than micromixing actuation using surface acoustic waves [27]. Lower actuation frequency could be useful for handling samples that contain living cells, as high frequency could induce undesired effects such as cell proliferation, differentiation, lysis etc. [28].

The mixing efficiency was studied by measuring the concentration of red pixels in the section of the microfluidic channel initially containing ethanol (blue) and the concentration of blue pixels in the microchannel section initially containing water. Within 30 s, a mixing efficiency of more than 75% over a volume of ~3 µL of the fluid (Figure 3D). This is comparable to some of the previously published results. For example, Liu et al. [29] have reported cavitation assisted mixing of 22 μL of the fluids in ~105 s, and for the system proposed by [30], the mixing efficiency is governed by the flow rate which is 8 μL/min. Both these values are close to what we observed, thus corroborating with the efficiency achieved through our micromixing technique. Continued operation of the micromixer produces agglomeration of non-covalently bound particulates from the coloring dye which interfered with mixing. However, for proof-of-concept preliminary demonstration of fluid mixing, our experiments show that efficient mixing of small fluid sample volumes can be achieved by integrating the piezo-actuated acoustic micromixer that generates micro-vortices through the controlled induction of cavitation bubbles.

### 3.3. Demonstration of On-Chip Optical Absorption

One of the major drawbacks of absorbance-based detection in microfluidics is that it is considered to decrease in the optical path length with reduced usage of the sample volumes, which could directly affect the sensitivity of the system [31]. Therefore, in order to ascertain whether our device configuration was sensitive enough to support real-time optical absorption measurements, we conducted a preliminary non-calibrated colorimetric assay. For this proof-of-concept experiment, we demonstrated the detection of glucose [32] in cell culture media, based on its reaction with Benedict’s reagent.

**Figure 4:**
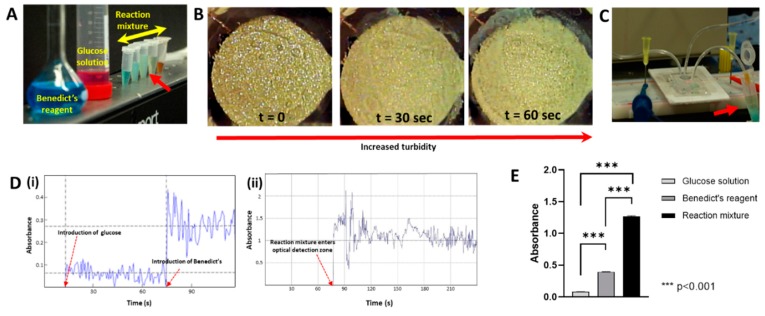
Implementation of Benedict’s reaction inside the optical microfluidic device for colorimetric detection of glucose in the presence of cell culture media. (**A**) Change in the color of the specimens for various concentrations of the glucose solutions, with the diluted samples showing shades of blue color. Reaction mixture upon heating turned to brick red color as expected. Arrow indicates the color obtained with undiluted glucose. (**B**) Variation, at different time points, in the turbidity of the reacting fluids (glucose solution and Benedict’s solution) upon piezo actuated mixing inside the microfluidic channel. (**C**) Reaction mixture ejected out of the microfluidic channel, indicating the same coloration as indicated in panel-A. (**D**) Real-time absorbance measurements at 635 nm wavelength of (i) glucose solution and the Benedict’s reagent and (ii) Reaction mixture upon entering the optical detection zone. (**E**) Comparison of the optical absorption values of the working fluids. The reaction mixture shows a significantly higher absorbance (*p* < 0.001) than both the glucose solution and the Benedict’s solution, due to the turbidity created upon mixing, thereby validating the colorimetric detection principle.

It is well known that glucose, a reducing sugar, induces the conversion of cupric ions (Cu^2+^) to cuprous form (Cu^+^) in the Benedict’s reagent [33]. This reaction brings about a change in color of the solution mixture, with the coloration being dependent upon the amount of glucose present. To firstly verify this principle, glucose-enriched media (hereafter referred to as glucose solution) was diluted to varying concentrations and mixed with the Benedict’s reagent. The color changes in the mixture samples are as shown in Figure 4A. The color observed for the reactant mixture for undiluted glucose solution is indicated with the arrow. Upon heating the mixture, the color of those reactants changed to dark red as expected.

Before implementing this colorimetric reaction inside the integrated optical microfluidic setup, baseline optical absorption values at 635 nm wavelength were recorded by passing the glucose solution and the Benedict’s reagent individually through the microfluidic channel. Thereafter, to initiate the reaction, the liquids were pipetted simultaneously into the microfluidic chamber, and the piezoacoustic micromixer was actuated. To the naked eye, the liquids initially appeared colorless upon entering the micromixing chamber (Figure 4B at t = 0). However, with continued piezo-actuation, mixing of the fluids causes an increase in the turbidity of the reaction mixture (Figure 4B).

The liquids were allowed to mix for 60 s, after which the piezo-actuation frequency was increased to drive the liquids under supercavitation [22] through the optical detection zone and finally out of the channels. Upon image processing, the color of this reaction mixture inside the microfluidic chamber (Figure 4B) and the color of the ejected reaction mixture (indicated in Figure 4C) was observed to be very close to the externally prepared reaction mixture indicated in Figure 4A, The results for the measurement of the colorimetric optical absorption is presented in Figure 4D. As expected, at 635 nm wavelength, the optical absorption of the blue colored Benedict’s reagent was higher than the phenol-red tagged glucose solution (Figure 4D(i)). The initial randomness in the optical absorbance signal for the reaction mixture (Figure 4D(ii)), is we believe, noise due to the flow of cavitation bubbles in the reaction mixture. The mean optical absorption of the reaction mixture was significantly higher (*p* < 0.001) than the both individual liquids, presumably due to the increased turbidity obtained during the reaction (Figure 4E). The optical absorbance value of reactants mixed inside the microfluidic channels was very close (<5% variation) to that of the reaction mixture externally prepared by manually vortexing the reacting fluids, thereby further confirming the homogeneity of the micromixing process. The experiment was repeated 3 times, and the results were found to be consistent. Thus, the results of this experiment demonstrates the implementation of optical absorption measurement, with our micromixing-enabled optical sensing setup.

### 3.4. Colorimetric Detection of Salivary Potassium

With the colorimetric detection feasibility well established through the proof-of-concept glucose detection experiments, we further explored the possibility of implementing our microfluidics-integrated optical detection platform for clinically relevant real-time biosensing applications. Potassium monitoring and level correction is a routine but critical procedure carried out at the hospitals for patients undergoing cardiac, renal or neurological related diagnoses. Altered potassium levels in the body under hyperkalemia or hypokalemia may cause arrhythmia, abnormal blood pressure, cramping, twitching or paralysis of muscles leading to the development of abnormal cardiac rhythms [34,35]. Since whole blood assays currently adopted in hospitals for potassium monitoring is painful and sometimes unsuccessful, we reasoned that salivary diagnostics of potassium could be far less traumatic and could generate rapid results when performed in a point-of-care setting. To validate this hypothesis, we tested whole saliva samples through colorimetric optical absorption, for the measurement of absolute salivary potassium concentration.

**Figure 5:**
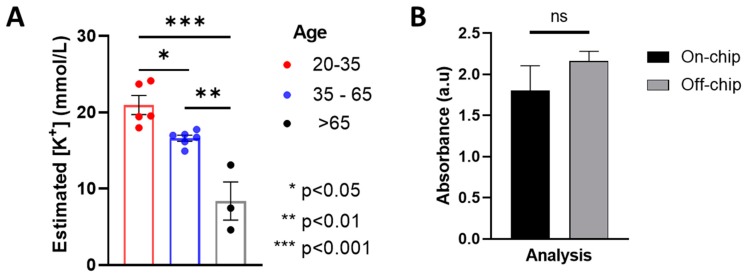
Results of the colorimetric optical absorbance detection of potassium from unstimulated whole saliva. (**A**) Measurement of estimated potassium concentration among people (15 volunteers) of different age groups indicating that older people (>65 y age) show a significantly lesser (*p* < 0.01) salivary potassium content than people in the age group of 35–65; and also significantly reduced potassium than people in 20–35 age group. In general people above the age of 35 show significantly lesser (*p* < 0.05) potassium than people under the age of 35. (**B**) Comparison of the optical absorbance values of salivary samples mixed with chromogenic agent measured on chip and off-chip (using an ultraviolet/visible (UV/Vis) spectrometer) shows no significant difference (*p* = 0.2643 with paired t-test) for the samples analyzed through the two measurement techniques.

The absorption measurements were conducted at 450 nm wavelength, as prescribed by the manufacturers of the potassium detection kit. Initial control experiments were conducted to measure the optical absorbance values of test samples with known potassium concentration. Unstimulated saliva samples were collected from random volunteers on the North Carolina A & T State University campus. Samples were collected from 3 volunteers above the age of 65 years, 6 volunteers in the age group of 35–65 years, and 5 student volunteers under the age of 35 years. The results of our colorimetric potassium concentration measurement from the collected saliva samples are presented in Figure 5A. The overall estimated mean concentration of salivary potassium was found to be well within the range of typical salivary potassium levels as reported earlier [36]. The mean unstimulated potassium concentration with people between the age of 13 and 35 has been reported to be ~21 mmol/L [37]. These observations match the data from our experimental results as well within 5% error, thereby validating our approach for a colorimetric optical absorbance-based salivary analysis method used herein.

However, contradicting observations have been made in literature regarding exact salivary potassium concentrations for patients with health conditions. We reasoned that there are several factors that could influence the salivary electrolyte concentrations, and a larger data set is required to make any robust comparison and inference using our device. However, the intent of the present work is far from delving into detailed biochemical analyses of saliva under different conditions. The objective of this work is to demonstrate the application of colorimetric sensing using microfluidic mixing for optical sensing. Therefore, in order to validate our optical measurements, we measured the optical absorbance of three random saliva samples treated with the chromogenic agents on-chip, and compared the results to the optical absorption of those samples measured externally using a standard spectrophotometer (Cary 6000i UV/Vis/NIR, Agilent, Santa Clara, CA, USA). The results shown in Figure 5B demonstrate no significant difference between the absorbance values measured through these two techniques, thereby validating our on-chip analysis approach. The time-dependent on-chip absorbance measurements and the absorbance spectrum obtained using the spectrophotometer for one of the samples is presented in Supplementary Figure S1A.

Thus, the results of our work show that the colorimetric optical absorption based biosensing principle with integrated micromixing can be readily applied to the lab-on-a-chip format for real-time analysis of metabolites from saliva to generate diagnostic results with a quick turn-around.

## 4. Discussion

Salivary diagnostics is being increasingly touted as a suitable alternative to whole blood assays. However, the study of salivary functions could also be challenging because of the high physiological variability of saliva in comparison with other bodily fluids [38]. However, given that the saliva samples can be obtained non-invasively, patients are much more likely to cooperate for frequent extraction of saliva, for performing assays regularly. We also observed this trend among the volunteers, who were unanimously relieved with the painless approach of collecting saliva samples for our experiments. Nevertheless, to exploit this convenience of salivary analysis, it is also equally important to develop a suitable automated point-of-care testing methodology that would enable rapid diagnostics to reduce the time and effort normally put into laboratory-based assays. The cumulative results of our present work demonstrates that the hybrid integrated optical biosensing principle proposed herein could be readily applied to potassium measurement from whole saliva samples for real-time point-of-care testing applications based on the principle of colorimetry.

The colorimetric optical absorption-based detection principle presented here is simple and can be easily extended to any other fluid sample. While the results of traditional point-of-care colorimetric assays reported to the naked eye (such as pregnancy tests or blood glucose tests), integrating smart-phones [39] for automated testings have lately been gaining huge popularity. But, the implementation of the colorimetric technique would not be effective unless uniform, controlled mixing of samples with the appropriate chromogenic agents is achieved. The acoustofluidic actuator thus lends a major advantage to this device for achieving the required homogenous sample mixing. The bulk acoustic wave micromixer configuration described in this work is simple and cost-effective to fabricate. For the device setup presented here, the ease of operation and feasibility of seamless hybrid integration with other complementary modules of the biosensor platform lend advantages to the bulk acoustic wave micromixer over the popularly used surface acoustic wave micromixing formats. However, mixing efficiency could be further improved by adopting simple modifications to the existing setup, such as incorporation of passive microfluidic elements, surface treatment resulting in increased hydrophobicity [40], and using acoustic index matching glues to improve piezoacoustic energy transfer. Our initial experiments showed that the cavitation-based acoustofluidic micromixing can be used with a wide variety of fluids with different physical properties such as miscibility, viscosities, vapor pressures etc. (Supplementary Figure S1B). While the present work focused only on cavitation-enabled fluid mixing, it was also observed during the initial characterization experiments that the cavitation phenomenon also induced other physical effects in the fluids. For example, it was possible to take advantage of the energy released from collapse of the cavitation bubbles for cleaning the surface of the microfluidic channels to get rid of non-specifically bound adherent particles [26]. This functionality could be extremely useful for conducting biological assays repeatedly without cross-contamination inside the microfluidic channels.

However, there are a few shortcomings to the present approach. Firstly, the device format in its present state does not enable the realization of cheap, disposable biosensors. But, since the device is sterilizable for repeated use, integrating light sources and sensor units on-chip along with suitable optoelectronic packaging could enable further help with the miniaturization of this device for developing a more cost-effective version. Secondly, optical absorption-based detection may hit the limits when conducting assays for measuring extremely low analyte concentrations in the samples. Despite the relatively poor sensitivity of microfluidic absorbance detection compared to fluorescence, the principle of working of optical absorption and its instrumentation simplicity gives it an advantage in applications requiring point-of-use analysis. Also, wherever applicable, it is possible to carry out a comprehensive study of time-dependent biochemical interactions using the optical absorption method. One can also take confidence from the fact that a number of absorbance-based microfluidic point-of-care products are commercially available [41]. Our device design also allows for implementing more than one optical sensing principle on the same platform. In an alternate waveguide arrangement to that used in the present work, the output waveguide was designed at an angle with respect to the inlet waveguide so that light is not directly coupled onto the output waveguide. This configuration, although not used in this work, could be useful for sensitive low absorption measurements or fluorescence-based optical detections where specific emission wavelengths are sensed spectroscopically without being saturated by the excitation wavelengths. Thus, overall our present work clearly demonstrates the feasibility of developing integrated microfluidic optical detection systems for rapid biosensing and real-time diagnostics, which could be incorporated into the next generation point-of-care devices.

## 5. Conclusions

Saliva is a non-invasive biofluid which is easy to collect, transport, and store. Because of its accessibility and connection to systemic diseases, saliva is one of the best candidates for the advancement of point-of-care medicine, where individuals are able to easily monitor their health status by integrating the salivary diagnostics with suitable biosensing systems. Our present work on the development of a hybrid integrated optical detection based biosensor with integrated micromixing further asserts that colorimetric salivary analysis can be conducted in real time in a dependable, noninvasive, simple, and rapid manner using whole, unprocessed saliva samples with the appropriate chromogenic agents, to screen for any required minerals and metabolite values Thus, this approach could come in handy for real-time point-of-care screening applications for monitoring high-risk hospitalized patients.

## Supplementary Materials

The following are available online at https://www.mdpi.com/2079-6374/9/2/73/s1: Figure S1: (**A**). (i) Time varying optical absorbance of a saliva sample mixed with chromogenic agent measured on-chip using the hybrid integrated device. The mean optical absorbance value was ~2.36 (ii) Optical absorbance measurement of the same sample measured using a UV/Vis spectrophotometer showing the absorbance value at 450 nm ~2.4. (**B**). Preparation of water in oil emulsion: Finer dispersion of water (blue) in the colorless oil medium throughout the chamber is observed with the actuation of the micromixer. Much longer micromixing time (~600 s) was required to achieve this, Figure S2: Enlarged versions of Figure 2E, Figure 4A,C.
