# Supplementary material for: Acoustofluidic Micromixing Enabled Hybrid Integrated Colorimetric Sensing, for Rapid Point-of-Care Measurement of Salivary Potassium
Source: Biosensors (Basel). 2019 May 28;9(2):73. doi: 10.3390/bios9020073 (PMC6628211; doi:10.3390/bios9020073)
Supplement: Supplementary file 1 [file biosensors-09-00073-s001.pdf]

Supplementary Figure

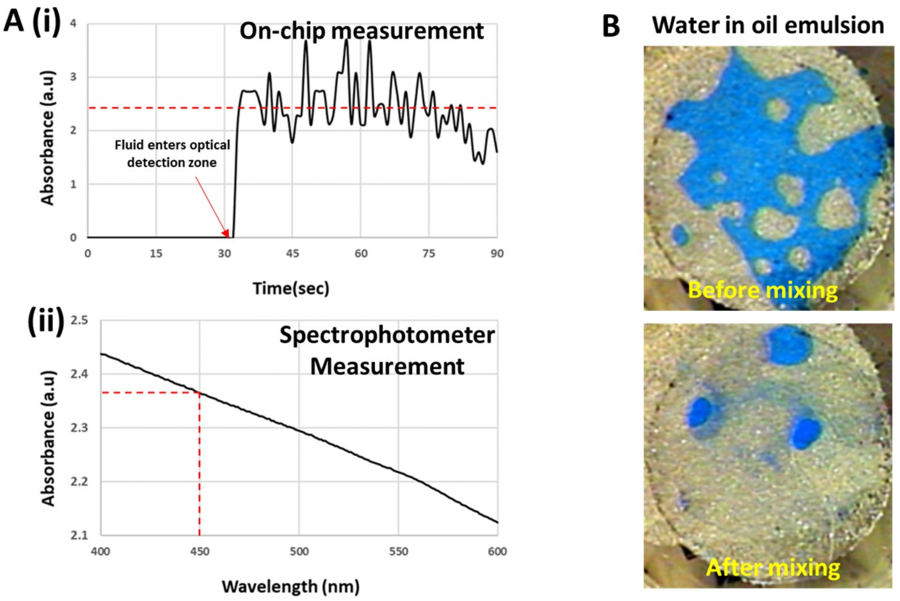

Figure 1. (**A**). (i) Time varying optical absorbance of a saliva sample mixed with chromogenic agent measured on-chip using the hybrid integrated device. The mean optical absorbance value was ~2.36 (ii) Optical absorbance measurement of the same sample measured using a UV/Vis spectrophotometer showing the absorbance value at 450 nm ~2.4. (**B**). Preparation of water in oil emulsion: Finer dispersion of water (blue) in the colorless oil medium throughout the chamber is observed with the actuation of the micromixer. Much longer micromixing time (~600 s) was required to achieve this.

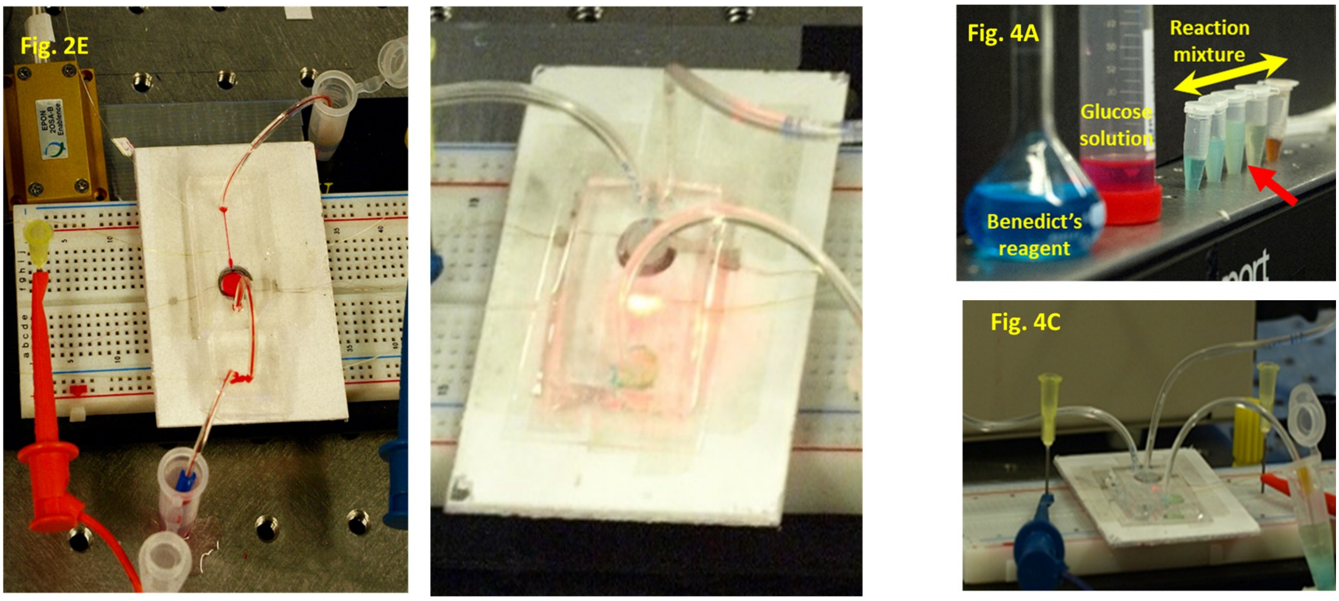

Figure S2. Enlarged versions of Figure 2E, Figure 4A and Figure 4C
